# Spinal fMRI Reveals Decreased Descending Inhibition during Secondary Mechanical Hyperalgesia

**DOI:** 10.1371/journal.pone.0112325

**Published:** 2014-11-05

**Authors:** Torge Rempe, Stephan Wolff, Christian Riedel, Ralf Baron, Patrick W. Stroman, Olav Jansen, Janne Gierthmühlen

**Affiliations:** 1 Dept of Neuroradiology, University Hospital of Kiel, Arnold-Heller-Strasse 3, Haus 41, 24105 Kiel, Germany; 2 Dept of Neurology, University Hospital of Kiel, Arnold-Heller-Strasse 3, Haus 41, 24105 Kiel, Germany; 3 Division of Neurological Pain Research and Therapy, University Hospital of Kiel, Arnold-Heller-Strasse 3, Haus 41, 24105 Kiel, Germany; 4 Centre for Neuroscience Studies, Dept of Diagnostic Radiology, Dept of Physics, 228 Botterell Hall, Queen’s University, Kingston, Ontario, Canada; University of Texas at Dallas, United States of America

## Abstract

Mechanical hyperalgesia is one distressing symptom of neuropathic pain which is explained by central sensitization of the nociceptive system. This sensitization can be induced experimentally with the heat/capsaicin sensitization model. The aim was to investigate and compare spinal and supraspinal activation patterns of identical mechanical stimulation before and after sensitization using functional spinal magnetic resonance imaging (spinal fMRI). Sixteen healthy subjects (6 female, 10 male, mean age 27.2±4.0 years) were investigated with mechanical stimulation of the C6 dermatome of the right forearm during spinal fMRI. Testing was always performed in the area outside of capsaicin application (i.e. area of secondary mechanical hyperalgesia). During slightly noxious mechanical stimulation before sensitization, activity was observed in ipsilateral dorsolateral pontine tegmentum (DLPT) which correlated with activity in ipsilateral spinal cord dorsal gray matter (dGM) suggesting activation of descending nociceptive inhibition. During secondary mechanical hyperalgesia, decreased activity was observed in bilateral DLPT, ipsilateral/midline rostral ventromedial medulla (RVM), and contralateral subnucleus reticularis dorsalis, which correlated with activity in ipsilateral dGM. Comparison of voxel-based activation patterns during mechanical stimulation before/after sensitization showed deactivations in RVM and activations in superficial ipsilateral dGM. This study revealed increased spinal activity and decreased activity in supraspinal centers involved in pain modulation (SRD, RVM, DLPT) during secondary mechanical hyperalgesia suggesting facilitation of nociception via decreased endogenous inhibition. Results should help prioritize approaches for further in vivo studies on pain processing and modulation in humans.

## Introduction

Mechanical pinprick hyperalgesia is a very distressing symptom presented by approximately 29% of neuropathic pain patients [1]. During a state of hyperalgesia, an already nociceptive stimulus is perceived as even more painful [1]. Secondary hyperalgesia develops in the uninjured area surrounding a nerve injury and is caused by central sensitization, i.e. by modulation of the spinal and supraspinal nociceptive system [2], [3]. It can be induced experimentally in healthy humans by the heat/capsaicin model [4].

Anatomically, the vast majority of the nociceptive second order neurons is located in the dorsal gray matter (dGM) of the spinal cord. From there they project to supraspinal nuclei in the brainstem and the thalamus before these afferent nociceptive impulses are transferred to further subcortical and cortical structures [5]. The nociceptive neurons in the spinal cord and in the brainstem have an important contribution in the transmission and modulation of pain. This knowledge is based mostly on animal experiments, observation of the effects of injury and disease as well as postmortem anatomical studies [3], [6], [7].

With the emergence of functional magnetic resonance imaging of the brain (fMRI) [8] and, to a lesser extent, of the brainstem [9], [10], non-invasive methods have become available to provide insight into human pain processing in vivo. To investigate pain processing on the spinal level and in the brainstem, spinal fMRI is of great interest, however, its utilization is limited because of significant technical challenges such as cerebro-spinal fluid-pulsations, breathing/swallowing-motions, and the spinal cord’s small cross-sectional dimension.

In order to address these challenges, recent spinal fMRI studies use a turbo spin-echo sequence with a relatively short echo time rather than the more conventional gradient echo imaging sequence sensitive to the blood oxygenation level-dependent (BOLD) effect [11]–[14]. It demonstrates BOLD contrast but also reveals a second contrast mechanism and important source of neuronal activity-related signal change in spinal fMRI termed “signal enhancement by extravascular water protons” (SEEP). This functionally induced signal is thought to originate from cellular swelling and thus changed extravascular water content due to increased intravascular pressure at sites of activity. This new technique is believed to localize sites of neuronal activity more precisely than conventional T_2_*-weighted gradient echo imaging sequences, which have rather poor field homogeneity [11]–[14].

However, spinal fMRI studies of human pain processing are still rare [15]–[17]. While spinal fMRI has recently been used successfully to demonstrate signal intensity changes in the spinal cord and brainstem during innocuous and noxious thermal stimulation and heat allodynia/hyperalgesia [18], there is still no spinal fMRI study that examines spinal and supraspinal changes in activity during secondary mechanical hyperalgesia up to this point.

Therefore, the aims were (A) to investigate spinal and supraspinal processes occurring as a consequence of mechanical stimulation and (B) to compare activation patterns of identical mechanical stimuli before and after induction of sensitization with the heat/capsaicin model in healthy subjects in order to investigate the specific pain-related components of secondary mechanical hyperalgesia. Within this study we could successfully demonstrate (A) increased activity in ipsilateral dGM after induction of hyperalgesia, (B) decreased activity in nociceptive regions of the brainstem and (C) a correlation between these supraspinal deactivations and activations of ipsilateral dGM during secondary mechanical hyperalgesia suggesting a facilitation of nociception via decreased descending endogenous inhibition.

## Materials and Methods

### Subjects

Sixteen right-handed [19] healthy volunteers (6 females, 10 males, mean age 27.2±4.0 years, range 23–32 years) were included in the study. All subjects were free of any acute or chronic pain conditions. Comorbidities such as diseases of the peripheral or central nervous system were ruled out. None of the subjects were on drugs that might have interfered with itch or pain sensations and flare responses. The study was in accordance with the Declaration of Helsinki and was approved by the Ethical Committee of the Faculty of Medicine at Christian-Albrechts-University of Kiel. Written informed consent was obtained from all participants.

### Psychophysics

**Figure 1:**
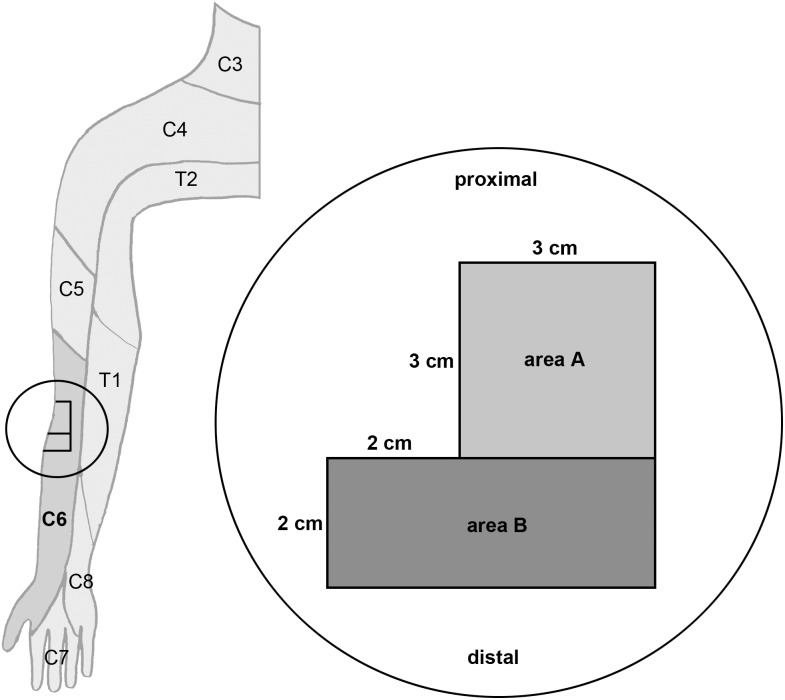
Areas of sensitization and stimulation on the right lateral volar forearm. For orientation, the schematic drawing on the left shows a dermatome map of the right upper extremity (redrawn and modified from [20]). The area of testing (marked by the circle) is situated on the right lateral volar forearm in the C6 dermatome. *Area A:* Site of sensitization with the heat/capsaicin model (3×3 cm square area 3 cm distal to the elbow in the C6 dermatome). *Area B:* Site of mechanical stimulation corresponding to the area of secondary mechanical hyperalgesia (2×5 cm area distal to area A).

On the right ( = ipsilateral) lateral volar forearm approximately 3 cm distal to the elbow in the C6 dermatome a 3×3 cm square area [area A, site of sensitization] and an adjacent 2×5 cm area directly distal to it [area B, for mechanical stimulation] were marked (figure 1).

### Experimental set-up

All subjects underwent two identical MRI sessions back-to-back on the same day with mechanical stimulation in the same area before and after sensitization.

#### fMRI data acquisition

All scans were performed in a 3T human MRI scanner (Philips Achieva 3T). For acquisition of functional image data, a half-Fourier, single-shot turbo spin-echo sequence with a phased-array head-neck receiver coil (TE = 38 ms, TR = 9000 ms, 288×144×20 mm FOV, 192×96 matrix) was used [17], [21]. At TE = 38 ms, this sequence is about equally sensitive to contributions of both BOLD and SEEP [14], [22], [23]. With this configuration 10 contiguous 2 mm thick sagittal slices were acquired, spanning from above the thalamus to below the C7/T1 intervertebral disc with a resulting voxel size of 1.5×1.5×2 mm^3^. To reduce sources of motion artefacts (e.g. heart, lungs and throat), a spatial saturation pulse was applied to the region anterior to the vertebral column.

#### Mechanical stimulation

In order to enable the stimulation of a larger dermatomal area (possibly leading to a higher signal in the spinal cord) mechanical stimulation was performed using a self-made brush consisting of 15 stiff von-Frey-hairs (166 mN). Care was taken that the site of stimulation was always within the area of secondary hyperalgesia [area B]. The mechanical stimulation paradigms consisted of 8 stimulation periods of 40 seconds alternating with eight baseline periods (64 volumes). After each session, subjects were asked to rate the stimulus intensity on the numerical rating scale (NRS, ranging from 0 to 10 with 0 representing “no pain” and 10 being the “maximum pain that can be imagined”) by verbal response.

#### Heat/Capsaicin sensitization model

The heat/capsaicin sensitization model was used to induce secondary mechanical hyperalgesia [4]. Therefore, area A was stimulated with a computer-controlled Peltier thermode (Medoc TSA-2001, Haifa, Israel) at 45°C for 5 minutes. Afterwards a gauze pad with 1 ml solution of 0.6% capsaicin in 45% ethanol was placed on area A for 30 minutes. During capsaicin-application, the subjects were asked once a minute for NRS ratings of perceived pain intensity and temperature sensation at the application site. The temperature sensation was quantified on the NRS with 0 representing “neutral temperature” and −10/+10 representing “the maximum cold/warmth that can be imagined”. After patch removal and at the end of the second fMRI block the borders of the area of punctate mechanical hyperalgesia were assessed by a stiff von-Frey-hair (166 mN). The dimensions of flare and mechanical hyperalgesia were then determined by the calculation: (D/2)×(d/2)×π (D = horizontal diameter, d = vertical diameters of the area) to assess the stability of the pain model throughout the experiment.

### Analysis and Statistics

For the analysis of the resulting 3D fMRI image data a general linear model (GLM) was used. The basis set consists of a boxcar model paradigm convolved with the tissue response function [12] and models of cardiac-related spinal cord motion as confounds [17], [21]. Its results demonstrate the weighting factors β1 (magnitude of the pattern matching the stimulation paradigm convolved with the tissue response function) and β0 (average voxel intensity). Corrections for bulk motion and a normalization to a consistent coordinate space of the brainstem and spinal cord were then performed as described previously [21]. A random-effects analysis by McGonigle et al. was used to determine combined group results [24]. This consists of the calculation of the mean and standard deviation of the ratio of β1/β0 representing the relative signal intensity response across studies. T values of >2.5 or <–2.5 were assumed as significant activity as they correspond with p<0.0075 [17]. Contrast calculations between mechanical stimulation before and after sensitization were performed on a voxel-by-voxel basis by the partial least squares (PLS) method [25]. A bootstrap ratio of ≥5 was chosen for differences between two contrasted responses to be significant [17].

Spinal cord segments adjacent to C6 were included in the analysis since (A) primary afferent fibers split up into longitudinal collaterals innervating the bordering segments and (B) slightly individual anatomical dermatome-borders with a coincidental stimulation of surrounding dermatomes (i.e. C5, T1) were respected.

Psychophysical data are presented as mean ± standard deviation (SD) unless otherwise specified. Wilcoxon matched pairs test was used for calculation of psychophysical intragroup differences and Spearman rank test for correlation calculations using the numbers of voxels (n_(v)_), signal change (Δ_(S)_) and % signal change (Δ_(S/S)_) across all volunteers provided by the fMRI data. P values <0.05 were considered to be statistically significant.

## Results

### Psychophysics

**Figure 2:**
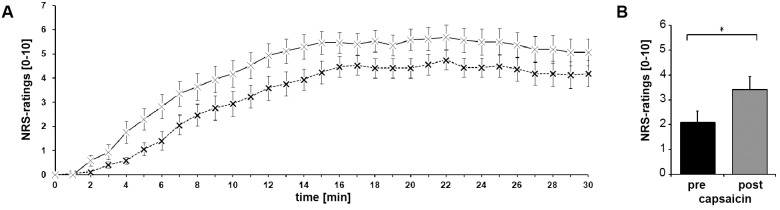
Psychophysical data. (A) Mean ratings of pain intensity (dashed line) and temperature perception (solid line) during capsaicin application. Capsaicin was applied at time 0. Mean ± standard error of the mean (SEM). (B) Mean pain ratings for the mechanical stimulus before and after application of capsaicin. Capsaicin induced secondary mechanical hyperalgesia. Mean ± SEM. *: p<0.05.

Capsaicin-application induced painful and warm sensations in all subjects (figure 2A). The capsaicin-induced flare decreased in size throughout testing (82.0±18.8 cm^2^ after patch removal vs. 67.0±16.8 cm^2^ at the end of testing, p = 0.002). However, the dimension of secondary mechanical hyperalgesia was stable (77.1±24.5 cm^2^ after removal of capsaicin vs. 89.5±35.7 cm^2^ at the end of scanning, p = 0,125) and included the area of stimulation [area B] at all times, thus ensuring the stability of the pain model throughout the experiment. Corresponding to mechanical hyperalgesia, the subjects’ mean ratings of pain intensity for the mechanical stimulus were higher compared to those before sensitization (3.4±2.2 vs. 2.1±1.8, p = 0.003; figure 2B).

### fMRI

#### Spinal group activation patterns and contrast maps

**Figure 3:**
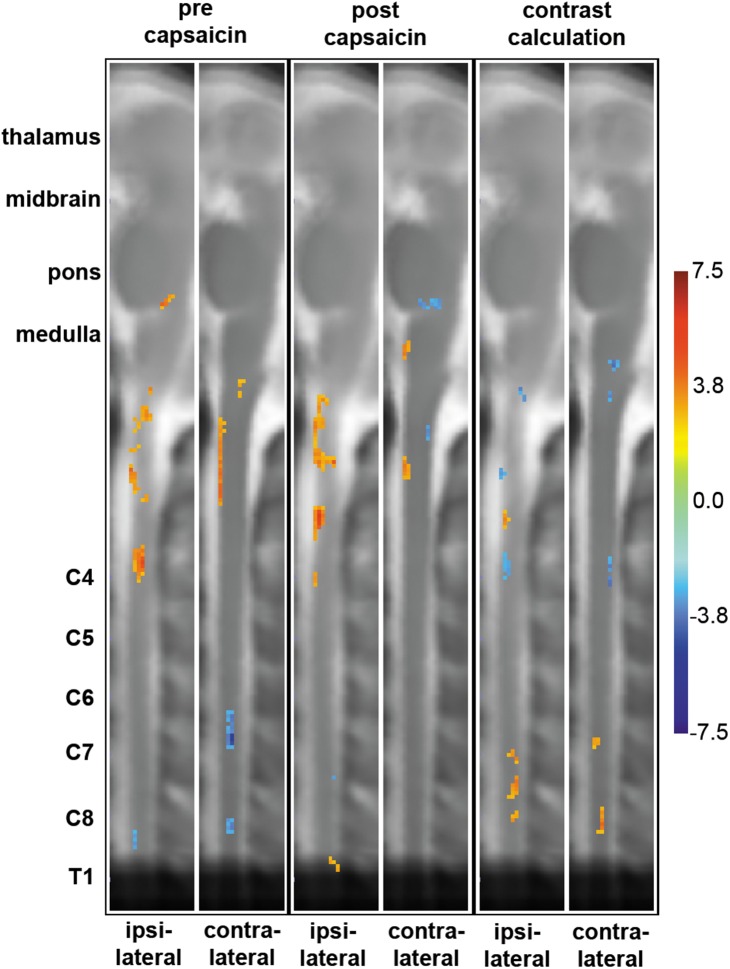
Sagittal slices of group activation patterns and contrast maps. Columns 1 to 4 show areas of activity across brain stem and cervical spinal cord before (1^st^ and 2^nd^ column) and after (3^rd^ and 4^th^ column) sensitization with the heat capsaicin model representing the significance (T-value) of each active voxel across the 16 subjects. Columns 5 and 6 show partial-least squares (PLS) results of contrast calculations on a voxel-by-voxel basis. The left column of each 2 columns (e.g. 1^st^, 3^rd^ and 5^th^) corresponds to the ipsilateral side of the stimulus, the right column to the contralateral side. The color bar on the right indicates the corresponding significance, i.e. T-value (columns 1–4) or bootstrap-ratio (columns 5–6) for each color.

**Figure 4:**
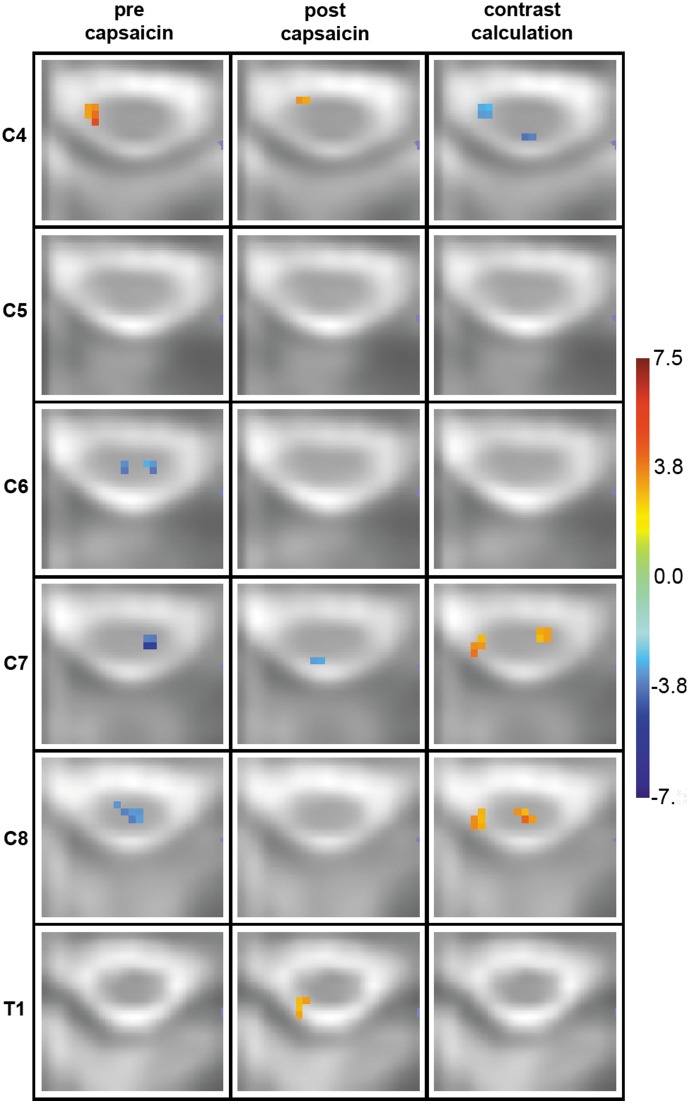
Spinal group activation patterns before (left column) and after (middle column) sensitization and contrast maps (right column). The transverse slices are in radiological orientation with the left side corresponding to the right body side and approximate the corresponding spinal cord segment for a rostral-caudal span from C4 to T1. They show spinal regions of signal intensity change before (left column) and after (middle column) sensitization with the heat/capsaicin model representing the significance (T-value) of each active voxel across the 16 subjects. The right column shows partial-least squares (PLS) results of contrast calculations on a voxel-by-voxel basis. The color bar in figure 3 indicates the corresponding significance, i.e. T-value (left and middle column) or bootstrap-ratio (right column) for each color. *Left Column:* Activations in ipsilateral vGM of C4. Deactivations in bilateral deep dGM of C6 and C8 and contralateral deep dGM of C7. *Middle column:* Ipsilateral activations in superficial dGM of T1 and vGM of C4. Deactivations in ipsilateral superficial dGM of C7. *Right column:* Activations in ipsilateral superficial dGM (C7, C8) and in contralateral vGM (C7) and deep dGM (C8). Deactivations in ipsilateral vGM and contralateral dGM of C4.

During mechanical stimulation prior to sensitization, spinal deactivations i.e. decreased signal intensity during stimulation were observed in deep dGM layers of C6 and C8 bilaterally and C7 contralaterally. After capsaicin exposure, mechanical stimulation in the area of secondary hyperalgesia lead to activations (increased signal intensity during stimulation) in T1 and deactivations in C7 in ipsilateral superficial dGM. Ventral activations were observed in ipsilateral ventral gray matter (vGM) of C4 before and after sensitization (figures 3, 4).

Activations in ipsilateral vGM of C7 correlated positively to the subjects’ NRS-ratings before (Δ_(S)_: R = 0.513; p = 0.042) and after sensitization (Δ_(S)_: R = 0.533; p = 0.041), i.e. the higher the perceived pain intensity, the higher were activations in vGM of C7.

Contrast calculations between mechanical stimulation before and after sensitization with the heat/capsaicin model revealed activations in superficial ipsilateral (C7, C8) dGM. The observed lateral deviation of superficial dGM-activations might arise from BOLD-contributions in signal change located in superficial draining veins [14]. Contralateral activations were observed in deep dGM of C8 and vGM of C7. Deactivations were found in C4 in ipsilateral vGM and contralateral dGM (figures 3, 4).

#### Supraspinal group activation patterns and contrast maps

**Figure 5:**
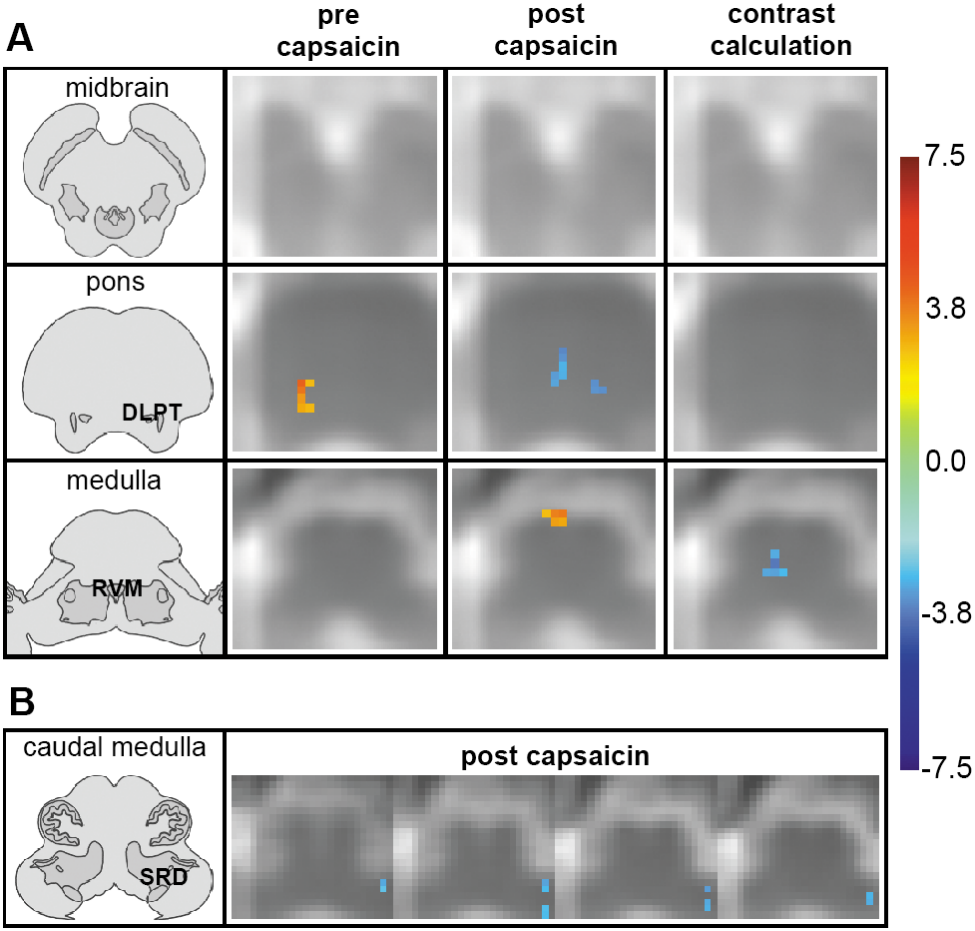
Supraspinal group activation patterns and contrast maps. Slices are in radiological orientation with the left side corresponding to the right body side. The color bar in figure 3 indicates the corresponding significance, i.e. T-value (combined group results pre/post capsaicin) or bootstrap-ratio (contrast calculation) for each color. Anatomical transverse sections on the left were modified from [26]. (A) The transverse slices approximate the corresponding brainstem region (midbrain, pons, medulla) for a rostral-caudal span. They show supraspinal regions of signal intensity change before (left column) and after (middle column) sensitization with the heat/capsaicin model representing the significance (T-value) of each active voxel across the 16 subjects. The right column shows partial-least squares (PLS) results of contrast calculations on a voxel-by-voxel basis. *Left column:* Activations in the ipsilateral DLPT (dorsolateral pontine tegmentum) during mechanical stimulation prior to application of capsaicin. *Middle column:* Deactivations in the contralateral DLPT and in the RVM (rostral ventromedial medulla) during secondary mechanical hyperalgesia (note that visible medial pontine deactivations are situated in the caudal pons/rostral medulla and therefore most likely correspond to the location of the RVM). *Right column:* Deactivations in the RVM. (B) The adjacent 1 mm thick transverse slices in consecutive arrangement located in the medulla show signal intensity changes during secondary mechanical hyperalgesia. Deactivations are observed in contralateral subnucleus reticularis dorsalis (SRD).

Prior to sensitization, supraspinal activity was observed in the ipsilateral dorsolateral pontine tegmentum (DLPT) (figure 5A). The activity in the ipsilateral dorsal pons correlated positively to activity in ipsilateral dGM of C4 (n_(v)_: R = 0.611; p = 0.012) and C5 (n_(v)_: R = 0.613; p = 0.012).

In contrast to measurements prior to sensitization, deactivations in contralateral DLPT were observed during secondary mechanical hyperalgesia (figure 5A). These deactivations correlated with activations in ipsilateral dGM of C5 (n_(v)_: R = 0.546; p = 0.035) and T1 (n_(v)_: R = 0.621; p = 0.012). Deactivations in the ipsilateral pons correlated with activations in ipsilateral dGM of C4 (n_(v)_: R = 0.595; p = 0.019) and T1 (n_(v)_: R = 0.555; p = 0.039; Δ_(S/S)_: R = –0.538; p = 0.047), i.e. the higher the deactivations in the dorsal pons, the higher were activations in ipsilateral dGM (note that R in n_(v)_ is positive because n_(deactivated voxels)_ correlates positively to n_(activated voxels)_ while Δ_(S/S)_ is negative because activations, i.e. positive Δ_(S/S)_-values are correlating to deactivations, i.e. negative Δ_(S/S)_-values).

After sensitization, deactivations were also visible in the ipsilateral and median caudal pons/rostral medulla most likely corresponding to the area of the rostral ventromedial medulla (RVM, figure 5A). Deactivations in the ipsilateral rostral ventral medulla correlated with ipsilateral dGM activations (C4: n_(v)_: R = 0.699; p = 0.004; Δ_(S)_: R = –0.827; p<0.001; Δ_(S/S)_: R = –0.770; p = 0.001; C6: Δ_(S)_: R = –0.537; p = 0.039). Deactivations in midline RVM correlated with deactivations in ipsilateral dGM (C5: n_(v)_: R = 0.556; p = 0.031; C7 =  n_(v)_: R = 0.599; p = 0.018; Δ_(S)_: R = 0.608; p = 0.016). Visible deactivations in the caudo-dorsal contralateral medulla corresponded to the localization of the subnucleus reticularis dorsalis (SRD, figure 5B) and correlated with spinal activations in ipsilateral dGM (C4: Δ_(S/S)_: R = –0.560; p = 0.030; C8: Δ_(S/S)_: R = –0.578; p = 0.024; T1 = n_(voxels)_: R = 0.554; p = 0.040).

Supraspinal calculations of contrast maps showed deactivations in the RVM (figure 5A).

## Discussion

The present study shows spinal and supraspinal signal intensity changes during mechanical stimulation and secondary mechanical hyperalgesia in healthy subjects. The main findings are (A) increased activity in ipsilateral dGM after induction of hyperalgesia, (B) decreased activity in supraspinal centers of pain processing and modulation (DLPT, RVM, SRD) and (C) a correlation between supraspinal deactivations and activations of ipsilateral dGM during secondary mechanical hyperalgesia induced by the heat/capsaicin model. Results suggest a facilitation of nociception via decreased descending endogenous inhibition during secondary mechanical hyperalgesia.

### Spinal cord

#### Ipsilateral dGM

Anatomically, the vast majority of primary nociceptive afferent fibers projects to the ipsilateral dGM. Therefore, application of noxious stimuli should lead to activation of projection neurons located in the marginal layer of the dorsal horn [27]. Supportingly, this study showed superficial dGM-activations during painful stimulation after sensitization. Animal experiments suggest a higher activation of spinal nociceptive projection neurons by the same noxious stimulus during experimental pain states [3], [6]. For example, Simone et al. showed an increase of monkey spinothalamic tract neuron responses to punctate mechanical stimuli during capsaicin-induced secondary hyperalgesia [2]. The current study is now the first one to reproduce such findings from animal experiments in human subjects in vivo as contrast maps revealed activations in superficial dGM that may correspond with increased activity during central sensitization.

Mechanical hyperalgesia and increased dGM activity can also be due to deficient inhibitory interneuron (ININ)-mediated inhibition of projection neurons [28], [29]. Interestingly, deactivations in deep dGM in C6 and C8 were observed prior to sensitization but disappeared during secondary mechanical hyperalgesia.

#### Contralateral dGM

Signal intensity changes were also observed in contralateral dGM during both conditions, consistent with a previous study using tactile stimuli [16]. These observations could be explained by primary afferent fibers ending in the contralateral dorsal horn [30] as well as by interneurons of ipsilateral dGM that cross the midline [17]. Our results also show differences during mechanical hyperalgesia compared to mechanical stimulation prior to sensitization suggesting a participation of the contralateral dGM in the development of central sensitization. It would be important for future studies to focus on such changes in contralateral dGM as contralateral somatosensory abnormalities have been observed in human unilateral neuropathic pain states [31].

#### vGM

Pain stimuli lead to withdrawal reflexes, i.e. the activation of ipsilateral motor neurons via spinal interneurons or supraspinal reticular nuclei [32]. Because mechanical stimuli were perceived as (slightly) painful, vGM-activity can be expected and was accordingly observed in both fMRI sessions. The positive correlation between the subjects’ NRS-ratings and the activity in ipsilateral vGM of C7 suggests that stronger pain stimuli lead to higher reflex answers. The C7 segment innervates both M. pronator teres and M. pronator quadratus. As the arm was held in a supine position throughout noxious stimulation, the activation of these muscles would be needed to escape the stimulus.

### Brainstem

#### DLPT

Animal testing shows that the DLPT plays a central role in the modulation of nociception. With its noradrenergic fibers to the dGM, it mediates a negative feedback loop triggered by noxious stimuli to prevent excessive pain sensation [7], [33]. This descending inhibition is for the most part mediated by the ipsilateral DLPT [34]. Accordingly, this study showed activations in the ipsilateral DLPT during painful mechanical stimulation prior to sensitization. Moreover these activations correlated with activations in ipsilateral dGM, possibly reflecting either an augmented activation of endogenous inhibition by higher activation of projection neurons [7] or a noradrenergic activation of spinal ININs (indirect inhibition of projection neurons) as it has been shown in animal studies [35]. Similarly, previous fMRI studies also showed activations of the DLPT during painful stimuli in healthy humans [15], [36].

An absence of this noradrenergic inhibition by the DLPT is thought to be a mechanism for the development of neuropathic pain [3], [37]. Interestingly, a bilateral decrease of DLPT-activation was seen during secondary mechanical hyperalgesia which correlated with ipsilateral dGM-activations. Compatibly, Becerra et al. showed bilaterally decreased DLPT-activity during mechanical hyperalgesia/allodynia in neuropathic pain patients [38]. Our results could thus correspond to a reduced descending noradrenergic inhibition with consecutive higher activation of spinal projection neurons.

Besides decreased inhibition, central sensitization is also thought to be mediated by coexisting excitatory descending facilitation [3], . Two earlier fMRI studies investigating secondary mechanical hyperalgesia demonstrated activity in rostral brainstem regions (midline-periaqueductal gray, contralateral cuneiform nucleus [10], contralateral mesencephalic pontine reticular formation [9]) that could be a correlate for excitatory nociceptive facilitation. Why did the current study not show these activations? Possible explanations could be overlapping processes of activation and simultaneous suppression of anti-nociceptive descending pathways in the very limited space of these brainstem nuclei or the different experimental set ups (MRI sequences or devices).

#### RVM

Based on current concepts of pain modulation, OFF-cells, a specific cell-type of the RVM, physiologically mediate antinociception [39], [40]. In the condition of central sensitization, this OFF-cell activity is decreased [41]. Compatibly, this study shows decreased activity in the RVM during secondary mechanical hyperalgesia. A deactivation of OFF-cells would lead to a decreased inhibition of spinal projection neurons. Supportingly, this study shows correlations between deactivation of ipsilateral RVM and activations in ipsilateral dGM during secondary mechanical hyperalgesia. However, it has to be kept in mind that spinal inhibition can also be caused by the activation of ININs [29]. The observed correlation between deactivations in median RVM and ipsilateral dGM could therefore correspond to decreased OFF-cell mediated activation of ININs during secondary mechanical hyperalgesia.

#### SRD

The SRD is believed to play a major role in the mechanism of diffuse noxious inhibitory control (DNIC) [42]. An absence of this control is thought to be a reason for the development of neuropathic pain [39]. Accordingly, this study shows deactivations in contralateral SRD during secondary mechanical hyperalgesia which correlated with activations in the ipsilateral dGM. This could correspond to a facilitation of nociception via decreased endogenous inhibition by SRD and consequently higher dGM-activations - a similar and additional mechanism to nociceptive modulation by DLPT and RVM.

### Limitations

Even though spin-echo spinal cord fMRI is a new reliable and non-invasive method to show functional processes in the spinal cord [15]–[18], its spatial resolution is still limited. Thus, an exact localization of the anatomical area corresponding to the observed activity is extremely difficult and can sometimes be only speculative. Furthermore, interpretation of results is complicated due to interconnections and often dichotomous roles of areas involved in pain processing/modulation, i.e. excitatory and inhibitory roles.

### Conclusion

Using spin-echo spinal cord fMRI, it was possible to investigate pain modulatory processes during secondary mechanical hyperalgesia in the brainstem and spinal cord of healthy subjects. This study is the first one to show an increase of ipsilateral dGM-activity during secondary mechanical hyperalgesia in human subjects in vivo. Furthermore, this study succeeds in showing decreased activity in areas of the brainstem that have been proven important for processes of central sensitization in animal experiments (DLPT, RVM, SRD). Moreover, those deactivations correlated with dGM-activity. With these findings, this study gives new insights in human pain processing in vivo. Since findings of animal experiments and results of previous fMRI studies could be reproduced, spin-echo spinal cord fMRI has been shown to be a reliable method for the examination of spinal and supraspinal pain processing and modulation. Herewith, it qualifies for further investigations including treatment of neuropathic pain.
